# Comparative transcriptome analysis reveals novel insights into transcriptional responses to phosphorus starvation in oil palm (*Elaeis guineensis*) root

**DOI:** 10.1186/s12863-021-00962-7

**Published:** 2021-02-05

**Authors:** Sze-Ling Kong, Siti Nor Akmar Abdullah, Chai-Ling Ho, Mohamed Hanafi bin Musa, Wan-Chin Yeap

**Affiliations:** 1 Laboratory of Sustainable Agronomy and Crop Protection, Institute of Plantation Studies, Universiti Putra Malaysia, 43400 UPM Serdang, Selangor Malaysia; 2 Department of Agriculture Technology, Faculty of Agriculture, University Putra Malaysia, 43400 Serdang, Selangor Malaysia; 3 Department of Cell and Molecular Biology, Faculty of Biotechnology and Biomolecular Sciences, University Putra Malaysia, 43400 Serdang, Selangor Malaysia; 4 Department of Land Management, Faculty of Agriculture, University Putra Malaysia, 43400 Serdang, Selangor Malaysia; 5 Sime Darby Technology Centre Sdn. Bhd., Block A, UPM-MTDC Technology Centre III, Lebuh Silikon, University Putra Malaysia, 43400 Serdang, Selangor Malaysia

**Keywords:** Phosphorus starvation, Transcriptome analysis, Oil palm, RNA-Seq, Differentially expressed genes, Pi-efficient

## Abstract

**Background:**

Phosphorus (P), in its orthophosphate form (Pi) is an essential macronutrient for oil palm early growth development in which Pi deficiency could later on be reflected in lower biomass production. Application of phosphate rock, a non-renewable resource has been the common practice to increase Pi accessibility and maintain crop productivity in Malaysia. However, high fixation rate of Pi in the native acidic tropical soils has led to excessive utilization of P fertilizers. This has caused serious environmental pollutions and cost increment. Even so, the Pi deficiency response mechanism in oil palm as one of the basic prerequisites for crop improvement remains largely unknown.

**Results:**

Using total RNA extracted from young roots as template, we performed a comparative transcriptome analysis on oil palm responding to 14d and 28d of Pi deprivation treatment and under adequate Pi supply. By using Illumina HiSeq4000 platform, RNA-Seq analysis was successfully conducted on 12 paired-end RNA-Seq libraries and generated more than 1.2 billion of clean reads in total. Transcript abundance estimated by fragments per kilobase per million fragments (FPKM) and differential expression analysis revealed 36 and 252 genes that are differentially regulated in Pi-starved roots at 14d and 28d, respectively. Genes possibly involved in regulating Pi homeostasis, nutrient uptake and transport, hormonal signaling and gene transcription were found among the differentially expressed genes.

**Conclusions:**

Our results showed that the molecular response mechanism underlying Pi starvation in oil palm is complexed and involved multilevel regulation of various sensing and signaling components. This contribution would generate valuable genomic resources in the effort to develop oil palm planting materials that possess Pi-use efficient trait through molecular manipulation and breeding programs.

**Supplementary Information:**

The online version contains supplementary material available at 10.1186/s12863-021-00962-7.

## Background

P is the second most limiting macronutrient for crop productivity after nitrogen. It acts as an essential constituent of nucleic acids important for storage and transfer of genetic information and as a structural element for a number of molecular compounds including ATP, ADP, phospholipid and coenzymes involved in energy transfer and physiological processes in plant cells [1]. P also plays a vital role in root development and in the whole reproductive process including fertilisation, seed set and fruit development [2]. P deficiency is thus expected to cause rapid and fundamental effects on crop growth and yield.

In order to adapt with the persistent Pi-limiting conditions, plants have evolved a variety of adaptive strategies, collectively known as Pi starvation responses (PSR) [3]. The implementation of these strategies requires sophisticated sensing and regulatory mechanisms that can integrate external and internal Pi status [4]. PSRs generally comprised of local and systemic responses. Local responses involve external Pi sensing and are regulated by local Pi status in monitoring root system architecture to enhance Pi acquisition whereas systemic or long distant responses are dependent on internal Pi concentration and include enhancement of Pi uptake, translocation and recycling of cytoplasmic Pi to maintain metabolic balance of P at the whole-plant level [3, 5]. A major part of the systemic responses in plant under Pi deprivation is regulated by PHOSPHATE STARVATION RESPONSE 1 (PHR1) and related transcription factors [6]. PHR1 mediated downstream Pi starvation-induced genes including *PHT1*, *PHF1*, *SPX*, *PAP* genes through binding to a P1BS *cis*-regulatory motif (GNATATNC) present in their promoters [7–11]. Apart from PHR1, other transcription factors have also been reported to be involved in transcriptional regulation of PSR such as WRKY45, WRKY75, WRKY42, OsMYB4P, OsMYB5P, ZAT6 and bHLH32 [12–18]. Most of these factors were identified in model plants, such as Arabidopsis and rice. In oil palm, however, only the high affinity phosphate transporter (*EgPHT1*) has been reported. Functional characterization of its promoter in homologous and heterologous model systems demonstrated that its activity is induced specifically in the roots under Pi starvation [19].

Oil palm (*Elaeis guineensis* Jacq.) is an economically important perennial crop in Malaysia which requires regular input of large amount of P fertilizer to sustain optimum oil yield. Starting from immature stage in the nursery, oil palm seedlings require intensive maintenance to attain maximum vegetative growth with well-balanced nutrition to produce high yielding mature oil palm trees. Sudradjat et al. [20] reported that the application of P fertilizer at the optimum rate of 4.24 g plant^− 1^ during six months at the main nursery linearly increased the total leave number and stem diameter of oil palm seedlings. Whilst reduction of leaf surface area, leaf expansion and leaf number were observed in Pi deficient oil palm seedlings which was later on reflected in lower biomass of fruit bunches produced at harvest [21]. In addition, young palms and seedlings that experienced insufficient Pi resulted in reduced plant height, stem girth and poor root development [2]. Phosphate rock is extensively used as P fertilizer for mature oil palm plantation in Malaysia, mainly attributing to its cheap price, rapid P dissolution and high P sorption capacity under rainfall and acidic soil conditions in the country [22, 23]. Predicted future scarcity of non-renewable rock Pi has been reflected by the US and China having stopped their export for strategic reasons [24]. Hence it is easy to foresee that the price of rock Pi will rise due to its increasing demand from all around the world and consequently the increase in oil palm production cost. With the soaring global demand for edible vegetable oils in conjunction with the growing world population, palm oil production will become increasingly important as it is expected to meet 65% of the 240 million tonnes demand by 2050 [25]. One of the approaches to reduce the impact of the predicted Pi source scarcity is improving the P-use efficiency of the crop itself through genetic means. However, knowledge on the molecular mechanism involved in modulation of Pi homeostasis in oil palm upon Pi deprivation as one of the basic prerequisites for genetic manipulation is quite limited.

In this study, we explored the transcriptome profiles activated by Pi-deficiency stress in oil palm seedling roots by transcriptome sequencing analysis. Comparison of the sequence-based expression profiles in oil palm seedling roots grown under sufficient Pi supply and Pi-depletion condition facilitated the identification of many genes whose expression are altered by Pi deficiency. These differentially regulated genes include various nutrient transporters, signalling components and transcription factors, which are believed to be involved in coordinating oil palm responses upon Pi scarcity. The findings reported in this work would increase our understanding of the signalling cascades involved in oil palm Pi-starvation responses and help in devising strategies to develop crops with better phosphate use efficiency which can minimize fertilizer input, manpower requirement in fertilizer management and environmental pollution and can ultimately help in decreasing production cost.

## Results

### Physiological responses to Pi deprivation

**Fig. 1:**
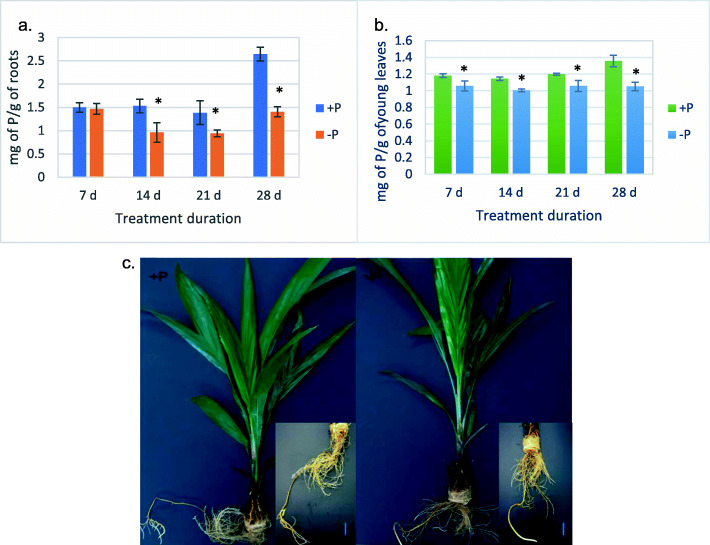
Physiological responses of oil palm seedlings to Pi-deprivation treatment. Total P concentration (mg g-1DW) of oil palm **a** roots and **b** young leaves under +P and -P conditions. The total P contents were assessed at 7d, 14d, 21d and 28d. Errors bars are standard deviation (*n* = 3). Asterisk indicates statistically significant (*p* < 0.05) differences between samples grown under +P and -P conditions. **c** Morphological phenotypes of oil palm seedlings after 28 days growth in +P and -P media. Bar = 2 cm

To assimilate the complex transcriptional responses in oil palm roots under Pi deficiency stress, we performed a time-course experiment, where 5 mon old seedlings were treated with Pi-deficient solution (0 mM Pi) for 7d, 14d, 21d and 28d. Total P content in young leaves and roots was measured to confirm the effectiveness of the Pi deprivation. In the roots, one-way ANOVA analysis indicated significant difference in the phosphate content of plants grown under the two conditions as early as 14 days after initiating the Pi treatment (*p* < 0.05) (Fig. 1a). Total P concentration in roots was significantly reduced (37.10%) after 14d of Pi-deficiency (−P) treatment and reaching more than 46% reduction after 28d. In contrast to the dramatic decline in roots, Pi deprivation for 28d only led to a 22.4% reduction in total P content in young leaves (Fig. 1b). Besides, the plants did not exhibit obvious growth difference in roots and leaves when observed by the naked eye until after 28d of Pi withdrawal. The plants in -P group possessed shorter primary root compared to Pi-sufficient (+P) group (Fig. 1c). Taken together, these results confirm the effectiveness of the Pi-deficiency treatment applied in the current study.

### Transcriptome response to Pi limitation

To examine the effects of Pi status on the transcriptome of oil palm seedlings roots, we selected two time points (14d and 28d) and used three biological replicates per condition for RNA-Seq, together with untreated controls representing a total of 12 libraries. By using Illumina HiSeq4000 platform, a dataset containing 202.6 gigabases and 1,350,329,088 clean reads (Q30 > 89%) was generated after excluding the low-quality reads. The error rate of all clean data per sample was controlled below 0.02%. Each of these samples comprised at least 99 million reads, of which more than 72% were uniquely mapped to the genome (Additional file 1). The total mapped reads for all samples were more than 70% and the multiple mapped reads were no more than 0.6%, which indicated high accuracy of the overall sequencing and the experiment is free from DNA contamination.

**Fig. 2:**
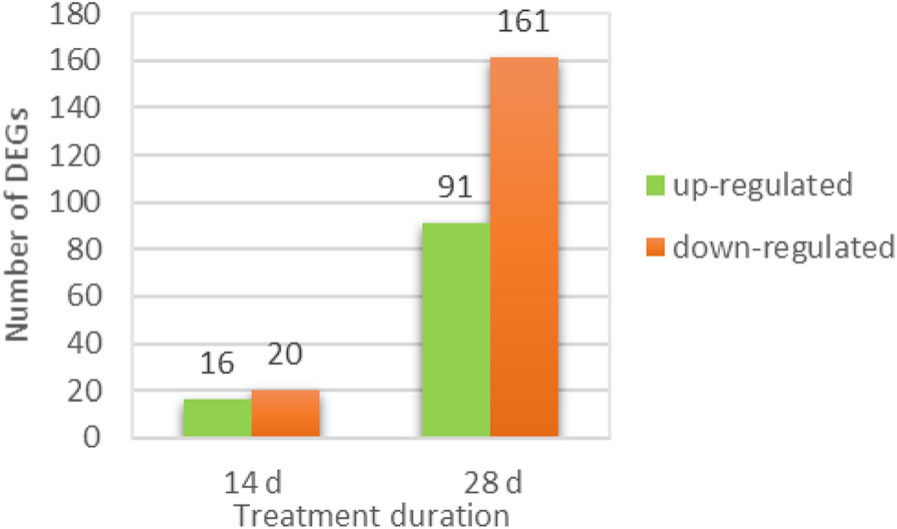
Number of DEGs identified through differential expression analysis on 14d and 28d transcriptome data

Differential expression analysis revealed a total of 36 and 252 differentially expressed genes (DEGs) in the oil palm roots after exposure to Pi deprivation for 14d and 28d, respectively. After 14d of -P treatment, 16 (44%) genes were up-regulated and 20 (56%) genes were down-regulated whereas 91 (36%) genes were up-regulated and 161 (64%) genes were down-regulated after 28d of -P treatment (Fig. 2). Venn diagram analysis shows that a total of seven DEGs; that is, four were up-regulated and three were down-regulated at both time points (Table 1). There was only one transcription factor (TF) encoding PCL1-like TF gene (105044363) being up-regulated at both 14d and 28d. The PCL1-like TF is required for generation of the clock oscillation in Arabidopsis [26]. The expression level of two 14–3-3-like proteins (105,041,596 and 105,037,590) was strongly repressed at both time points.

**Table 1:** DEGs that co-expressed at both 14d and 28d (− 1 < log_2_ fold change > 1; q-value < 0.05)

DEG accession no.	T1 vs C1		T2 vs C2		Annotation	Arabidopsis homologue accession no.
	Log_**2**_ FC	q-value	Log_**2**_ FC	q-value		
105,041,362	2.644	3.71e-3	2.475	1.25e-3	Uncharacterized protein	OAO90986
105,038,152	2.389	6.13e-3	2.209	2.08e-2	Aquaporin NIP6–1-like protein	NP_178191
105,044,363	2.335	1.01e-2	1.999	6.90e-3	Transcription factor PCL1-like	NP_001030823
105,053,650	2.020	2.14e-2	2.441	2.21e-2	Photosystem I reaction center subunit IV	NP_179616
105,051,924	−2.663	1.01e-2	−1.941	1.97e-2	Chlorophyllide a oxygenase	NP_175088
105,041,596	−8.990	3.12e-2	−2.785	1.86e-3	14–3-3-like protein GF14 omega	AAA96253
105,037,590	−10.442	2.27e-2	−2.988	3.36e-7	14–3-3-like protein 16R	NP_565176

*T1* denotes -P group at 14d, *C1* denotes +P group at 14d, *T1 vs C1* denotes -P/+P comparison at 14d, *T2* denotes -P group at 28d, *C2* denotes +P group at 28d, *T2 vs C2* denotes -P/+P comparison at 28d. FC denotes fold change

### DEGs in response to Pi deprivation

In this study, six candidate genes related to Pi signalling and homeostasis were detected at 28d but not at 14d (Table 2). Proteins harbouring the SPX domain have been proven to act in Pi sensing and adaptations to Pi deprivation in plants [27]. There were three genes encoding SPX domain-containing proteins (105,054,157, 105,058,610 and 105,047,822) being up-regulated in Pi-starved roots. Purple acid phosphatases (PAPs) are a type of APase involved in P scavenging and utilization in plants [28]. Here, one APase gene (105048024) and two PAP genes (105,055,553 and 105,056,384) were found to be induced by Pi deficiency.

**Table 2:** List of DEGs possibly involved in Pi homeostasis at 28d Pi deprivation

DEG accession no.	Log_**2**_ FC	q-value	Annotation	Arabidopsis homologue accession no.
105,054,157	2.620	9.92e-6	SPX-MFS domain-containing protein	NP_567674
105,058,610	2.039	3.37e-3	SPX domain-containing protein 5	NP_182038
105,047,822	1.878	1.32e-2	SPX domain-containing protein 1	NP_197515
105,055,553	2.890	4.16e-3	Purple acid phosphatase 23	NP_193106
105,056,384	1.563	2.69e-2	Purple acid phosphatase 3	NP_172923
105,048,024	2.621	7.64e-4	Acid phosphatase 1	NP_194655

It is well known that macro and micro-elements are co-ordinately integrated with each other in response to fluctuation in their availability during growing condition, ranging from over excess to extreme deficiency [29]. Hence, it is not surprising that transporters for nutrients other than phosphate were also found to be responsive to Pi deprivation. Numerous transporter genes were identified in this study but most of them were down-regulated including sulfate transporter, boron transporter and nitrate transporter (Table 3). Meanwhile, some genes belonging to the same transporter family were differentially regulated upon Pi deficiency. For example, zinc (Zn) transporter 6 and Zn transporter 4 were up-regulated and down-regulated at 28d, respectively. A similar situation also occurred to members of the aquaporin gene family. All these results are implying the requisite for adjustment among multiple plant nutrients while oil palm plants encounter low Pi stress.

**Table 3:** Transporter genes that were differentially expressed in Pi-deficient roots

DEG accession no.	T1 vs C1		T2 vs C2		Annotation	Arabidopsis homologue accession no.
	Log_**2**_ FC	q-value	Log_**2**_ FC	q-value		
105,038,152	2.389	6.13e-3	2.208	2.08e-2	Aquaporin NIP6–1-like protein	NP_178191
105,044,284	−2.030	1.01e-2	–	–	Aquaporin PIP2–2	NP_181254
105,048,091	–	–	−2.001	3.63e-3	Aquaporin NIP1–1-like protein	NP_193626
105,057,126	–	–	1.949	1.73e-2	Zinc transporter 6	NP_180569
105,059,891	–	–	−2.041	5.14e-3	Zinc transporter 4	NP_187881
105,052,257	–	–	−1.417	2.43e-2	Sodium-coupled neutral amino acid transporter 5	NP_191179
105,048,115	–	–	−1.645	4.07e-2	Mitochondrial dicarboxylate/tricarboxylate transporter	NP_197477
105,043,754	–	–	−1.781	4.39e-2	Probable sulfate transporter 3.5	NP_568377
105,058,392	–	–	−1.959	4.17e-2	Sulfate transporter 1.3-like	NP_001319061
105,058,391	–	–	−3.114	8.88e-5	Sulfate transporter 1.3-like	NP_001319061
105,040,206	–	–	−1.970	3.37e-3	Boron transporter 4-like	NP_001319010
105,050,303	–	–	−2.049	4.39e-2	Nucleobase-ascorbate transporter 6-like	NP_176211
105,046,978	–	–	−2.121	1.56e-2	Equilibrative nucleotide transporter 3-like	NP_001329797
105,033,813	–	–	−2.843	2.41e-2	High affinity nitrate transporter 2.4-like	NP_200885
105,045,799	–	–	−1.365	3.50e-2	Probable sugar phosphate/ phosphate translocator	NP_187740
105,042,295	–	–	−2.081	3.43e-2	Cation/calcium exchanger 1-like	NP_197288

A total of 22 putative TFs within 13 families were annotated from the DEG list using the plant TF database PlantTFDB version 4.0 (http://planttfdb.cbi.pku.edu.cn/) (Table 4). Among these, the proteins belonging to the MYB and G2-like family made up the two most abundant DEGs. In plants, most of the identified MYB family proteins are associated with Pi starvation regulatory mechanism [30]. All three TFs belonging to the MYB family exhibited attenuated expression patterns at 14d. Meanwhile half of the MYB proteins were up-regulated at 28d. Three TFs belonging to the G2-like family were detected from the DEG list. Remarkably, two G2-like TFs containing MYB-CC domain (105,050,046 and 105,058,550) were inversely regulated at 28d (one up-regulated and the other down-regulated). Besides, both bHLH family TFs (105,048,562 and 105,051,179) were found to be suppressed at 28d in which the latter encoding for a FER-LIKE IRON DEFICIENCY_INDUCED (FIT) TF. FIT TF is recognized as the key player in Fe homeostasis by regulating the expression of iron deficiency responsive genes [31]. Plant hormones assist in plant responses to nutrient limitation by mediating nutrient signalling and plant growth and development [32]. In this study, three genes encoding TFs potentially involved in hormone signal transduction were found to be responsive to Pi deprivation stress. Two ethylene signalling-related genes (105,059,334 and 105,046,219) were up-regulated at 28d and categorized into different TF families, AP2/ERF and EIL respectively. In addition, expression of a putative scarecrow-like protein (105032345), a member of GRAS family, was highly induced in roots under low Pi stress. GRAS gene family members are involved in diverse elemental processes of plant growth and development, ranging from gibberellin acid signalling, radial root patterning and phytochrome signalling [33].

**Table 4:** Genes encoding transcription factors that were differentially expressed in Pi-deficient oil palm seedling roots

DEG accession no.	T1 vs C1		T2 vs C2		Annotation	Arabidopsis homologue accession no.	Family
	Log_**2**_ FC	q-value	Log_**2**_ FC	q-value			
105,055,259	−4.154	2.42e-6	–	–	Protein CCA1	NP_198542	MYB
105,058,870	−2.804	2.61e-2	–	–	Protein CCA1-like	AAS09985	MYB
105,059,546	−1.945	2.61e-2	–	–	Protein LHY-like	BAH19541	MYB
105,040,489	–	–	1.711	1.39e-2	MYBS3	NP_200495	MYB
105,045,660	–	–	1.891	1.46e-2	AS1-like	NP_181299	MYB
105,054,880	–	–	−2.365	9.05e-3	LAF1	NP_200039	MYB
105,059,220	–	–	−2.599	3.76e-2	LAF1-like	NP_194286	MYB
105,037,502	–	–	−2.458	3.43e-2	WRKY72	OAO89951	WRKY
105,044,363	2.335	1.01e-2	1.999	6.90e-3	PCL1-like protein	NP_001030823	G2-like
105,050,046	–	–	1.752	1.58e-2	PHL7-like protein	NP_178216	G2-like
105,058,550	–	–	−1.759	2.59e-2	PHL8-like protein	NP_001118567	G2-like
105,048,652	–	–	−1.642	4.83e-2	bHLH94-like	NP_177366	bHLH
105,051,179	–	–	−5.188	4.43e-2	FER-LIKE IRON DEFICIENCY-INDUCED TF (FIT)	NP_850114	bHLH
105,059,334	–	–	1.652	3.14e-2	Dehydration-responsive element-binding protein 1C	ABV27118	AP2/ERF
105,046,219	–	–	∞	9.05e-3	Ethylene-insensitive 3-like (EIL) protein	NP_188713	EIL
105,032,345	–	–	2.178	4.77e-3	Scarecrow-like protein 15	NP_195389	GRAS
105,049,070	–	–	−2.411	1.86e-2	NAC domain-containing protein 21/22-like	NP_175997	NAC
105,058,898	–	–	1.654	2.24e-2	Zinc finger protein NUTCRACKER-like	AAL91203	C2H2
105,049,340	–	–	2.071	4.20e-2	Zinc finger CCCH domain-containing protein 40-like	NP_563788	C3H
105,034,688	–	–	−2.309	2.85e-3	Protein TIFY 9-like	NP_568287	TIFY
105,041,208	–	–	−2.510	5.87e-5	LOB domain-containing protein 40-like	NP_566175	LOB
105,059,465	–	–	−2.800	5.28e-3	Homeobox-leucine zipper protein HAT4-like	NP_193476	HB

In order to evaluate the potential functions of these identified DEGs, all of them were subjected to GO functional enrichment analysis. The top 30 most enriched GO terms were listed and grouped into three categories, namely biological processes, molecular functions and cellular components (Additional file 2). In the biological process ontology, “carbohydrate metabolic process” and “single-organism process” were the most highly represented terms in roots at 14d and 28d, respectively. Regarding molecular function, the dominant term at 14d was “hormone activity”. Meanwhile two terms (“molecular function regulator” and “enzyme regulator activity”) accounted for the majority of the molecular function ontology at 28d. The results showed that DEGs at both time points were enriched in the molecular function and biological process categories, suggesting that molecular functions and biological processes play important roles in Pi-starvation responses of oil palm.

KEGG pathway enrichment analysis was conducted to illustrate the DEG-associated pathways involved in Pi-starvation responses. There were 14 significant enriched KEGG pathways identified at 28d with q-value less than 0.05 (Table 5). Among these 14 pathways, all seven DEGs associated with the pathway of “protein processing in endoplasmic reticulum” were up-regulated. Whereas the expression of DEGs associated with the pathways (“sulfur metabolism”, “nitrogen metabolism”, “taurine and hypotaurine metabolism”, “ascorbate and aldarate metabolism”, “alanine, aspartate and glutamate metabolism”, “monobactam biosynthesis” and “selenocompound metabolism”) were all repressed after Pi-starvation for 28d in roots.

**Table 5:** List of significantly enriched pathways at 28d with q-value < 0.05

Term	Pathway ID	Input number	Background number	q-value
Metabolic pathways	ath01100	44	1910	6.54e-8
Sulfur metabolism	ath00920	5	41	8.94e-4
Biosynthesis of secondary metabolites	ath01110	23	1076	8.94e-4
Taurine and hypotaurine metabolism	ath00430	3	14	4.85e-3
Ascorbate and aldarate metabolism	ath00053	4	41	5.82e-3
Pyruvate metabolism	ath00620	5	85	8.59e-3
Protein processing in endoplasmic reticulum	ath04141	7	212	1.70e-2
Phenylpropanoid biosynthesis	ath00940	6	157	1.70e-2
Glyoxylate and dicarboxylate metabolism	ath00630	4	74	2.50e-2
Pentose and glucuronate interconversions	ath00040	4	81	3.05e-2
Nitrogen metabolism	ath00910	3	42	3.10e-2
Monobactam biosynthesis	ath00261	2	14	3.50e-2
Alanine, aspartate and glutamate metabolism	ath00250	3	48	3.72e-2
Selenocompound metabolism	ath00450	2	18	4.65e-2

### MapMan analysis

**Fig. 3:**
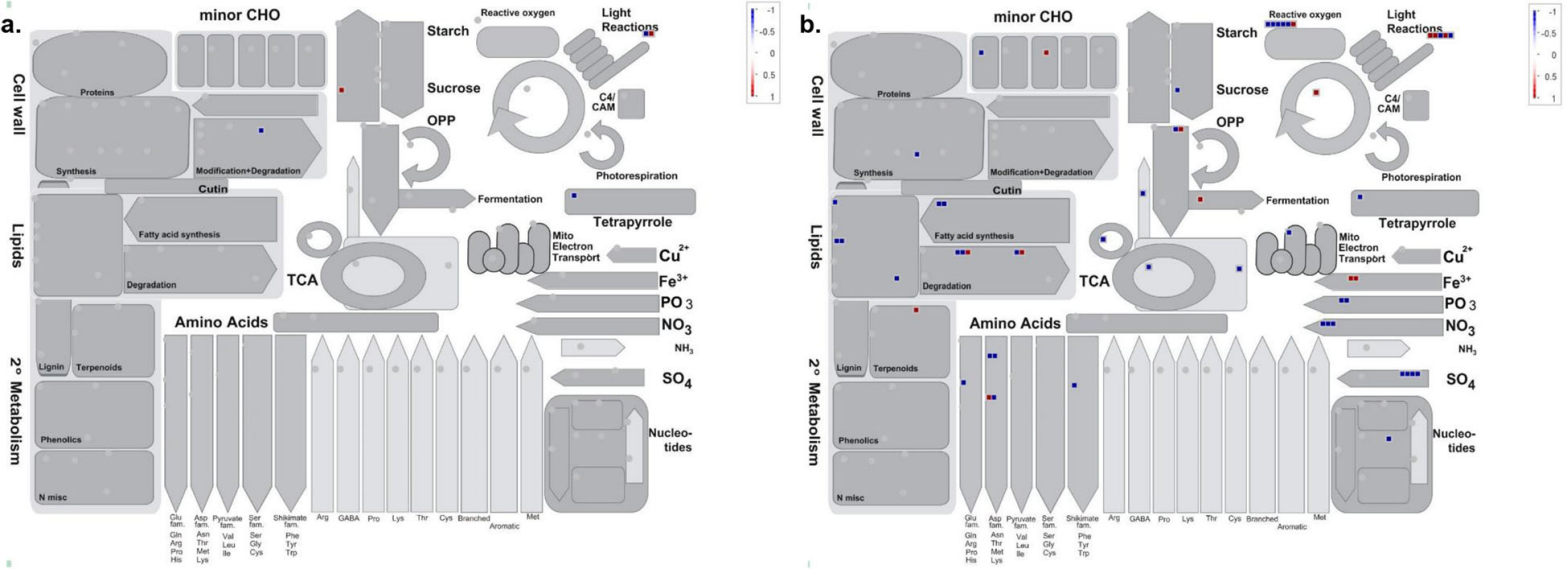
MapMan metabolism overview maps depicting differences in DEGs transcript levels after **a** 14d and **b** 28d of Pi deprivation treatment. Individual genes are represented by small squares. The colour key represents the value of log_2_ fold change between +P and -P group. Blue represents down-regulated transcripts and red represents up-regulated transcripts

To investigate the metabolic pathways involved in response to phosphate deficiency stress, we analysed the metabolism overview associated with 36 and 252 DEGs detected at 14d and 28d, respectively (Fig. 3). For 14d, five DEGs were assigned into four different metabolism pathways including photosynthesis, cell wall metabolism, coenzyme metabolism and carbohydrate metabolism (Fig. 3a; Additional file 3). Among 252 DEGs found at 28d, 55 genes were classified into several diverse pathways; 11 for lipid metabolism including lipid degradation and glycerolipid biosynthesis; 11 for nutrient uptake including sulfur and nitrogen assimilation; six for amino acid metabolism; six for photosynthesis, six for redox homeostasis; five for carbohydrate metabolism and five for cellular respiration; as well as several others involved in regulation of this stress such as secondary metabolism and cell wall organisation (Fig. 3b; Additional file 3). These results implied that distinct metabolic pathways were being triggered as part of the stress responses after 14d and 28d Pi-starvation treatment in oil palm.

### RNA-Seq validation by qRT-PCR

**Fig. 4:**
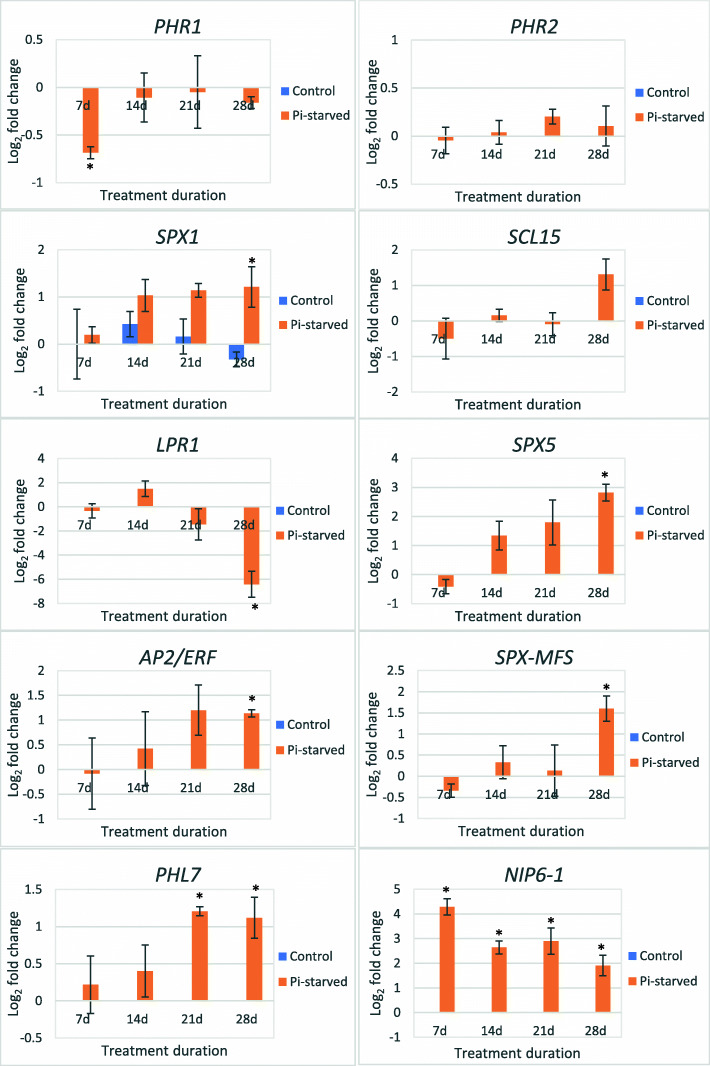
Examination of the DEGs expression profiles at four different time points using qRT-PCR analysis. Ten candidate genes were selected for validation using oil palm seedling roots treated under Pi sufficient (control) and deficient (Pi-starved) conditions for 7d, 14d, 21d and 28d. The y-axis represents log_2_ fold-change of the relative expression level normalized with all three normalization factors, *NADH5*, *GRAS* and *β-actin* and the x-axis represented the treatment duration. The data shown are the mean log_2_ fold change of the relative expression level of three biological replicates with standard error. Asterisks indicate significant difference between control and Pi-starved treatments in the Student t-test (*p* < 0.05). NIP6–1: Aquaporin NIP6–1 like protein (105038152); SPX5: SPX domain-containing protein 5 (105058610); SPX-MFS: SPX-MFS domain-containing protein (105054157); SPX1: SPX domain-containing protein 1 (105047822); PHL7: PHL7-like protein (105050046); AP2/ERF: dehydration-responsive element-binding protein 1C (105059334); LPR1: multicopper oxidase LPR1-like protein (105041188); SCL15: Scarecrow-like protein 15 (105032345)

To validate the deep sequencing results, the expression profiles of 10 transcripts were examined by real-time quantitative PCR on oil palm young roots exposed to Pi deficiency stress for 7d, 14d, 21d and 28d. Among the 10 genes, eight were shortlisted from the DEG list obtained from RNA-Seq analysis while two (*PHR1* and *PHR2*) were selected as they have been extensively reported as key transcription factors that orchestrate the Pi-starvation regulations in other plant species. The results showed that the expression of eight DEGs showed similar trend (seven up-regulated and one down-regulated at 28d) to those of the RNA-Seq, suggesting the reliability of the RNA-Seq results (Table 6). Meanwhile, the expression of *PHR1* and *PHR2* only experienced scarce fluctuation throughout the 28d of Pi deprivation treatment (Fig. 4). Interestingly, the expression pattern is different between *PHR1* and *PHR2* in which *PHR1* was being repressed as compared with the marginal increment in *PHR2* transcription level. On the contrary, the expression of *PHR1-like 7* (*PHL7*) was significantly induced at 21d and 28d. *NIP6–1,* the DEG that was expressed at both 14d and 28d and included in the qRT-PCR analysis, was substantially up-regulated at all time points indicating that this gene possibly plays an important role in Pi-starvation regulatory mechanism in oil palm seedling roots.

**Table 6:** Validation of RNA-Seq data using qRT-PCR analysis

DEG accession no.	Arabidopsis homologue accession no.	RNA-Seq		qRT-PCR	
		14d	28d	14d	28d
105,038,152	NP_178191	2.389	2.208	2.638 ± 0.22	1.907 ± 0.41
105,058,610	NP_182038	–	2.039	–	2.818 ± 0.28
105,054,157	NP_567674	–	2.620	–	1.601 ± 0.29
105,047,822	NP_197515	–	1.878	–	1.212 ± 0.43
105,050,046	NP_178216	–	1.752	–	1.118 ± 0.28
105,059,334	ABV27118	–	1.652	–	1.136 ± 0.07
105,041,188	OAP17281	–	−5.726	–	−6.413 ± 1.07
105,032,345	NP_195389	–	2.178	–	1.308 ± 0.44

RNA-Seq data was presented in the values of log_2_ fold change with q-value < 0.05. Data of qPCR are expressed as mean log_2_ fold change of the relative expression level of three biological replicates with standard error. The fold expressions of each gene in qRT-PCR analysis were normalized by all three reference genes; *GRAS*, *NADH5* and *ß-actin* expression levels. *NIP6–1*: Aquaporin NIP6–1 like protein (105038152); *SPX5*: SPX domain-containing protein 5 (105058610); *SPX-MFS*: SPX-MFS domain-containing protein (105054157); *SPX1*: SPX domain-containing protein 1 (105047822); *PHL7*: PHL7-like protein (105050046); *AP2/ERF*: dehydration-responsive element-binding protein 1C (105059334); *LPR1*: multicopper oxidase LPR1-like protein (105041188); *SCL15*: scarecrow-like protein 15 (105032345)

## Discussion

Plants frequently encounter low Pi availability in soils and have thus established a series of adaptive morphological, physiological and biochemical strategies to cope with Pi deficiency. Modification of root growth and architecture is a well-documented morphological response to Pi starvation including reduction of primary root length [34]. In addition, a decline of P concentration in Pi-deprived plants has been reported in other plant species under similar -P treatment [35–38]. In contrast to the distinct declination in young roots, the total P content in young leaves was consistent throughout the treatment period. This probably was caused by Pi homeostasis in the plant where re-translocation of Pi from older leaves to younger leaves occurred during Pi starvation [39]. Interestingly, the total P content in young roots was slightly increased (9%) at 28d compared to 21d. This could be the adaptive response by plant under severe phosphate deficiency where roots will become a sink tissue rather than a source tissue in order to enhance root proliferation and soil exploration [40]. Thus, 14d and 28d were selected for investigating the early and late responses of oil palm roots under Pi starvation stress through RNA-Seq.

Understanding the underlying molecular mechanisms is important for developing P-use efficient crop cultivars to optimize crop yield with less investment of P fertilizer. In recent years, RNA-Seq has been extensively employed for transcriptome studies of numerous economically important crop plants under Pi deficit condition [41–44]. To the best of our knowledge, this is the first study reporting the transcriptomic responses of oil palm seedlings roots to Pi deficiency using RNA-Seq approach and identified two different groups of PSR genes at two time points. Under similar treatment, the number of DEGs identified at 28d was nearly seven-fold higher than that at 14d, implying that the plants responded more actively and dramatically as the time of stress increased (Fig. 2). Similarly, short term Pi deprivation also resulted in considerably lower number of DEGs in rice and barley [37, 45]. As compared to 14d, significantly higher number of DEGs participated in assorted biological processes including nutrient transport, lipid metabolism and amino acid metabolism at 28d based on the metabolism overview obtained from MapMan analysis. These DEGs at 28d could be identified as ‘late’ genes that alter the physiology and metabolism of plants upon prolonged Pi deficiency [46].

14–3-3 proteins are a family of phosphoserine-binding proteins that are able to recognize and bind to the well-defined phosphorylated motifs of a number of target proteins via direct interaction. Their association to a phosphorylated target can eventually alter its subcellular localization, protein stability, enzyme activities and /or protein-protein interactions [47]. *GRF9* encoding a 14–3-3 isoform has demonstrated its role in the regulation of metabolic pathways during Pi-starvation responses in Arabidopsis [48]. Moreover, 14–3-3 proteins were found to modulate plasma membrane H^+^-ATPase functioning in Pi acquisition and enzyme activities involved in carbohydrate and nitrogen metabolism, which is one of the plant adaptations to low Pi stress [49, 50]. In tomato, the expression of 14–3-3 proteins were spatial and temporally regulated in response to Pi limitation [51]. Xu et al. [52] also reported that two 14–3-3 isoforms, *TFT6* and *TFT7* that were differentially expressed in tomato plants displayed distinct roles in acclimation to Pi deficiency. In this study, Pi deficiency was found to suppress all four 14–3-3 proteins (105,041,596, 105,037,590, 105,041,440 and 105,037,838) expression in oil palm seedling roots. Hence, the role of 14–3-3 proteins in Pi homeostasis deserves further studies since 14–3-3 s have been shown to be involved in various cellular processes including plant hormone signalling and biosynthesis [53].

A highly conserved PHR1-mediated signalling cascade has been well-documented in Arabidopsis and rice [7, 54]. Although AtPHR1 in Arabidopsis and its functional equivalent, OsPHR2 in rice have been demonstrated as the central player in coordinating various transcriptional regulations in response to Pi starvation, the expression of both transcripts were irrespective to Pi fluctuation [55, 56]. Nonetheless, the expression profiles for both *PHR1* and *PHR2* in oil palm were relatively stable throughout the 28d of Pi deprivation treatment as revealed in the qRT-PCR analysis. In Arabidopsis, PHR1 was shown to act redundantly with other members in the MYB-CC family, PHL1, PHL2, PHL3 and PHL4 in modulating plant transcriptional responses to Pi scarcity. Besides, the expression levels of *AtPHL2* and *AtPHL3* were also shown to be positively triggered by Pi starvation while the others were not affected by external Pi levels [7, 57, 58]. Meanwhile, two genes encoding PHL7 and PHL8 TFs were differentially regulated in oil palm seedling roots at 28d. By possessing a common MYB domain and a coiled-coil domain, both proteins might also play a key role in controlling oil palm transcriptional responses to Pi deficiency similar to their orthologue in Arabidopsis. Moreover, *PHL7* was significantly induced starting from 21d to 28d in Pi-starved oil palm root tissues as revealed in the qPCR analysis. The distinctive transcription pattern of these two TFs under low Pi stress suggested that they may be regulated by different molecular components.

In recent years, the importance of SPX domain-containing proteins in plant Pi homeostasis including sensing, signalling, and transport of Pi have been illustrated [59, 60]. SPX proteins refer to proteins exclusively harbouring the conservative SPX (SYG1/PHO81/XPR1) domain [61]. Previous studies have demonstrated that the activities of PHR1 are negatively regulated by SPX1 in Arabidopsis or SPX1, SPX2 and SPX4 in rice in a Pi-dependent manner [62–64]. In the presence of Pi, SPX protein binds to PHR1 at high affinity and restricted its binding to P1BS *cis*-element. Conversely, the binding affinity of the SPX-PHR1/2 complex declined in the absence of Pi and PHR1 is released to activate the transcription of downstream Pi-starvation induced genes [62]. In oil palm seedling roots, two SPX-domain containing genes (105,058,610 and 105,047,822) were low Pi inducible. This scenario was in agreement with those observed in Pi deficiency transcriptome analysis of other plant species [37, 38]. Moreover, a SPX-MFS domain-containing gene (105054157) was also significantly up-regulated at 28d. Protein harbouring SPX-MFS domain was designated as a member of PHOSPHATE TRANSPORTER 5 family (PHT5) and involved in vacuolar Pi sequestration to maintain cytoplasmic Pi equilibrium in the cell [65–67]. In plant cells, vacuoles seem to play a dual role as source and sink of Pi and changing of Pi concentration in cytosol or vacuole could acts as signal to activate PSR pathway [40]. Thus, this SPX-MFS domain-containing gene is probably involved in modulation of Pi homeostasis in oil palm seedling roots after experiencing prolonged Pi scarcity stress.

Induction and secretion of intracellular and/or extracellular APases are considered to be an important acclimation strategy for plant tolerance under low Pi environment which has been documented in diverse crop plants [68, 69]. PAPs represented the largest class of plant APases that could be secreted into the rhizosphere to hydrolyse organic P compounds whereas the intracellular PAPs could facilitate the Pi remobilization from internal reservoir. With the presence of P1BS motifs in their promoters, PAP genes are positively controlled by the PHR1-mediated Pi starvation signalling pathway [70, 71]. Accretion of APases transcripts was commonly reported in several recent transcriptomic studies involving Pi-starved soybean, maize and banana [41, 72, 73]. Therefore, it is expected that three APases genes were positively stimulated at 28d. Transgenic plants overexpressing PAP gene depicted increased APase activities, leading to the enhanced use of external organic P sources, higher plant biomass and eventually improved plant growth under Pi limitation [11, 74]. Hence, these differentially regulated PAP genes deserve further studies with regards to their roles in Pi scavenging and recycling.

Among the identified DEGs, many genes are possibly involved in transportation of water, sulfate, zinc and other nutrients other than Pi. Pi deprivation has been shown to diminish plants root hydraulic conductivity and causes disruption of water transport [75]. Three aquaporin encoding genes were differentially modulated in Pi deprived oil palm seedling roots. A putative aquaporin *PIP2–2* (105044284) was down-regulated at 14d of -Pi treatment and the same expression profile was also reported for all six PIP genes in Pi-starved sheep grass [76]. Meanwhile, the expression of a candidate aquaporin *NIP6–1* gene (105038152) was constantly up-regulated in this study with the highest expression level at 7d as revealed in qRT-PCR analysis. NIPs may play a role in plant stress responses since the activity of these proteins would be enhanced by phosphorylation under stress conditions. Transgenic plants overexpressing aquaporins showed higher tolerance to environment stresses [77]. Therefore, it would be intriguing to determine the contribution of this up-regulated aquaporin *NIP6–1* in oil palm Pi stress regulation mechanism through functional characterization.

Pi scarcity has profound impacts on diverse metabolic pathways as well as on transcription control. Such transcription reprogramming is expected to assist plants in accommodating to Pi deficiency and altering metabolism to ensure durability. After 28d growth in Pi-depleted media, several sulfate transporter genes were found to be down-regulated in oil palm seedling roots. SULTR1;3, a phloem-specific sulfate transporter is responsible for source-sink redistribution of sulfate while SULTR3;5 performs in synergy with SULTR2;1 in root-to-shoot transfer of sulfate [78, 79]. Conjointly with that, five DEGs (two ATP sulfurylase genes, two APS reductase genes and one cytochrome C gene) involved in sulfur metabolism were also being repressed. ATP-sulfurylase catalysed the first step in sulfate metabolism through adenylylation reaction to forms 5′-adenylylsulfate (APS) which subsequently undergo reduction assimilation carried out by the enzyme APS reductase [80]. Hence, it is conceivable that sulfate subcellular and inter-organ translocation and assimilation process was attenuated by prolonged Pi deficiency stress in oil palm seedling roots. Besides, Pi deprivation also exhibits repression effects on the expression of Fe-deficiency-induced Fe acquisition genes [81–83]. Even though no Fe acquisition related gene being listed as DEG in this study, there was a bHLH transcription factor, FIT being detected. Colangelo and Guerinot [84] had demonstrated the importance of FIT in the regulation of two major players in iron uptake system, FRO2 and IRT1 at the transcriptional level and protein accumulation level respectively. In oil palm, the abundance of FIT transcripts was tremendously down-regulated at 28d which is consistent with the same down-regulated expression profile reported for Pi deficient Arabidopsis roots [85]. This could likely be one of the rebalancing responses in plants to impede iron uptake in order to avoid the frequently observed iron overload in plants under low Pi stress [85, 86].

The critical roles of TFs in modulating the transcription alterations of their downstream target genes when plants encounter Pi scarcity situation has been well reviewed [5, 87, 88]. Among the Pi-starvation responsive DEGs in the current study, 22 TFs belonging to several families consisting of MYB, WRKY, AP2/ERF, zing finger proteins and bHLH were identified with MYB family members accounting the majority. MYB TFs can be divided into different classes based on the number and types of the conserved MYB repeats present in their DNA-binding domain [89]. Here, the seven identified MYB TFs can be divided into two classes, namely R2R3-MYB (105,045,660, 105,054,880 and 105,059,220) and 1R-MYB (105,055,259, 105,058,870, 105,059,546 and 105,040,489). In fact, the three G2-like family TFs (105,044,363, 105,050,046 and 105,058,550) were also grouped into 1R-MYB family as they possess a single MYB repeat at their N-terminus. In addition, Pi-deficiency was also shown to alter the expression of several ERF genes, a group of AP2 domain-containing TFs that are involved in ethylene-responsive genes regulation in Arabidopsis [90]. Liu et al. [91] also established the crucial role of ethylene-insensitive 3 (EIN3) and ethylene-insensitive 3-like (EIL1) in coordinating the ethylene-mediated Pi-starvation responses through activation of PHR1 transcription. Therefore, the two Pi-deficiency induced ethylene-related TFs (105,059,334 and 105,046,219) may contribute in Pi-deficiency regulation mechanism in oil palm although their exact roles need to be further testified.

## Conclusion

In summary, our RNA-Seq analysis was successfully conducted on 12 paired-end RNA libraries and the results unveiled genome wide expression profile of oil palm seedling roots in response to Pi deprivation stress. Analysis of the transcriptome datasets identified transcripts that encode diverse transcription factors, transporters and signalling components from a total of 288 DEGs. Most of the identified DEGs were consistent with the previously reported studies on Pi-starved plants including the induction of several SPX-domain containing genes and APases genes. Nevertheless, we also discovered some candidate genes such as *PHL7*, *NIP6–1* and 14–3-3 genes which possibly took part in the Pi-deficiency modulation and acclimation in oil palm. Transcripts, involved in sulfate remobilization and assimilation process and iron uptake, were also found to be repressed by prolonged Pi scarcity stress in oil palm. The results suggested an intricate signalling and regulation cascade governing Pi homeostasis in oil palm involving multiple metabolism pathways. These findings have improved our understanding of the Pi homeostasis in oil palm root at the molecular level and laid a solid basis for further functional characterization of those candidate genes associated with Pi-use efficiency trait in oil palm.

## Methods

### Plant materials and treatment

A total of 48 three months old oil palm seedlings (DxP GH500) were purchased from Sime Darby Seeds & Agricultural Services Sdn. Bhd and grown hydroponically for eight weeks on Pi sufficient medium which contained 5.77 mM KNO_3_, 4.25 mM Ca (NO_3_)_2_, 2.1 mM MgSO_4_, 0.2 mM FeNaEDTA, 36 μM MnSO_4_, 27 μM H_3_BO_3_, 1.56 μM CuSO_4_, 0.3 μM (NH_4_)_6_Mo_7_O_24_ and 1.5 mM ZnSO_4_, with 1.93 mM KH_2_PO_4._ They were then separated into two groups (+P and -P). In +P group, the plants were continuously supplied with Pi sufficient nutrient solution whereas in -P group, the plants were supplied with nutrient solution without KH_2_PO_4_ to induce Pi deficiency stress. KH_2_PO_4_ was replaced with K_2_SO_4_ in -P condition to maintain the concentration of K. Young roots and young leaves of both groups were harvested at the following time points: 7d, 14d, 21d and 28d.

### Quantification of total P concentration

The fresh weight of the harvested young roots and young leaves were measured and then dried at 70 °C until constant dry weight was obtained. Dried tissues (0.25 g) were then converted to ash by burning in a furnace at 300 °C for an hour and subsequently at 500 °C for 4 h. After overnight cooling, the ash was removed from the furnace and subjected to acid digestion, then quantified using an auto-analyzer (QuikChem, Series 8000, Lachat Instruments Inc., USA). The one-way ANOVA was used to determine whether there are any statistically significant differences between the means of the (+P) and (−P) samples. The mean comparison was carried out using Duncan Multiple Range Test at α = 0.05% using SAS software version 9.4.

### Total RNA isolation and RNA-Seq analysis

Three biological replicates from 14d and 28d root tissues in both treatment groups were used for RNA-Seq analysis. Total RNA was extracted following a modified CTAB method as described [92]. DNase I treatment was carried out according to the manufacturer’s instructions (Fermentas, USA). The integrity and quality of the extracted total RNA were assessed using Qubit fluorometer (Thermo Fisher Scientific, USA) and Agilent 2100 Bioanalyzer (Agilent Technologies, USA).

RNA-Seq was performed on an Illumina HiSeq4000 platform (Novogene, Singapore) to generate 150 bp paired-end reads. After trimming the poor-quality reads and adaptor sequences, the clean reads were then mapped onto the reference genome of *Elaeis guineensis* available in NCBI database under accession number PRJNA192219, using TopHat2 algorithm with a maximum of 2 mismatches. Transcript abundance in FPKM was estimated using HTSeq with union mode. For differential expression analysis, DESeq with a corrected *p*-values < 0.05 was employed. All DEGs were then subjected to the GO term overrepresentation test using GOseq [93]. Regulatory pathways were investigated by matching DEGs to putative orthologs in the Kyoto Encyclopedia of Genes and Genomes (KEGG) protein database (www.genome.jp/dbget/) [94].

### MapMan analysis

For metabolic pathway analysis, oil palm transcripts were annotated and classified into MapMan BINs using the MapMan Mercator4 version 2.0 (https://plabipd.de/portal/mercator4) [95]. The functional category analysis of DEGs was performed by MapMan version 3.6.0 [96].

### Quantitative RT-PCR analysis

First-strand cDNAs were synthesized from 1 μg of total RNA using Maxima First Strand cDNA synthesis kit (Thermo Fisher Scientific Inc., Waltham, MA). The qRT-PCR was carried out on StepOne Plus (Applied Biosystems, Foster city, CA, USA) with Fast SYBR Green Master Mix (Applied Biosystems, Foster city, CA, USA) according to manufacturer’s instructions. After each run, a dissociation curve was generated to verify the amplification specificity. Three biological replicates were included for both control and treatment group and tested in triplicate. No template control was included in each run. PCR efficiencies for each primer set were analysed by amplifying serial dilutions of a mixture of all cDNA from all samples. Only primer sets that amplified with efficiency above 85% and exhibited a single and specific peak in dissociation analysis were shortlisted. All selected primer sets were listed in the Additional file 4. Relative expression levels of all ten selected DEGs were calculated using delta-delta Ct method after normalized to three internal controls (*NADH5*, *GRAS* and *β-actin*).

## Supplementary Information

**Additional file 1: Table S1.** Summary statistics for RNA-Seq output of 12 paired-end libraries.

**Additional file 2: Figure S1.** The 30 most enriched GO classification for (a) 14d and (b) 28d. The y-axis shows the GO terms and the x-axis shows the number of differential expression genes. Different colours are assigned to biological process, cellular component and molecular function respectively.

**Additional file 3: Table S2.** Summary of MapMan analysis associated with the phosphate deficiency DEGs in this study.

**Additional file 4: Table S3.** List of primers used for quantitative gene expression analysis in oil palm seedlings.

## Data Availability

The data of sequenced mRNA are available in the National Center of Biotechnology Information (NCBI) under the accession number PRJNA673667.

## Abbreviations

ADP: Adenosine diphosphate
ANOVA: Analysis of variance
AP2/ERF: APETALA2 and ethylene-responsive element binding proteins
APases: Acid phosphatases
APS: 5′-adenylylsulfate
ATP: Adenosine triphosphate
bHLH: Basic helix-loop helix
DEG: Differentially expressed gene
EIL: EIN3-like
EIN3: Ethylene-insensitive 3
FIT: FER-LIKE IRON DEFICIENCY-INDUCED transcription factor
FPKM: Fragments per kilobase of transcript per million fragments mapped
GO: Gene ontology
KEGG: Kyoto Encyclopedia of Genes and Genomes
P: Phosphorus
PAP: Purple acid phosphatase
PHF1: Phosphate Transporter Traffic Facilitator 1
PHL: PHR1-like
PHR1: Phosphate Starvation Response 1
PHT: Phosphate Transporter
Pi: Phosphorus orthophosphate
PSR: Phosphate starvation response
qRT-PCR: Quantitative real-time polymerase chain reaction
RNA-Seq: mRNA sequencing
SPX: SYG1/PHO81/XPR1
TF: Transcription factor
Zn: Zinc
