# Supplementary material for: Comparative transcriptome analysis reveals novel insights into transcriptional responses to phosphorus starvation in oil palm (Elaeis guineensis) root
Source: BMC Genom Data. 2021 Feb 5;22:6. doi: 10.1186/s12863-021-00962-7 (PMC7863428; doi:10.1186/s12863-021-00962-7)
Supplement: Supplementary file 1 — Additional file 1: Table S1. Summary statistics for RNA-Seq output of 12 paired-end libraries. [file 12863_2021_962_MOESM1_ESM.pdf]

Additional file 1: Table S1. Summary statistics for RNA-Seq output of 12 paired-end libraries.

**Sample name**	**C1_1**	**C1_2**	**C1_3**	**T1_1**	**T1_2**	**T1_3**	**C2_1**	**C2_2**	**C2_3**	**T2_1**	**T2_2**	**T2_3**
**Total clean reads (million)**	108	105	106	102	114	116	111	115	100	161	99	107
**Q30 (%)**	89.12	89.64	92.78	92.63	92.59	92.43	92.59	92.72	91.71	90.84	90.22	90.85
**GC (%)**	49.40	48.95	48.86	48.80	49.07	49.01	48.73	49.18	49.12	49.23	49.40	50.83
**Total mapped reads (%)**	77.01	78.03	83.69	82.52	83.14	83.18	82.21	83.07	83.15	79.61	79.54	73.45
**Uniquely mapped reads (%)**	76.60	77.65	83.33	82.16	82.78	82.79	81.82	82.68	82.77	79.20	79.17	72.86

C1 denotes control group (+P) for 14d; T1 denotes Pi-starved group (-P) for 14d; C2 denotes +P for 28d; T2 denotes -P for 28d. Each group comprised of three biological replicates.
