# Supplementary material for: Comparative transcriptome analysis reveals novel insights into transcriptional responses to phosphorus starvation in oil palm (Elaeis guineensis) root
Source: BMC Genom Data. 2021 Feb 5;22:6. doi: 10.1186/s12863-021-00962-7 (PMC7863428; doi:10.1186/s12863-021-00962-7)
Supplement: Supplementary file 2 — Additional file 2: Figure S1. The 30 most enriched GO classification for (a) 14d and (b) 28d. The y-axis shows the GO terms and the x-axis shows the number of differential expression genes. Different colours are assigned to biological process, cellular component and molecular function respectively. [file 12863_2021_962_MOESM2_ESM.pdf]

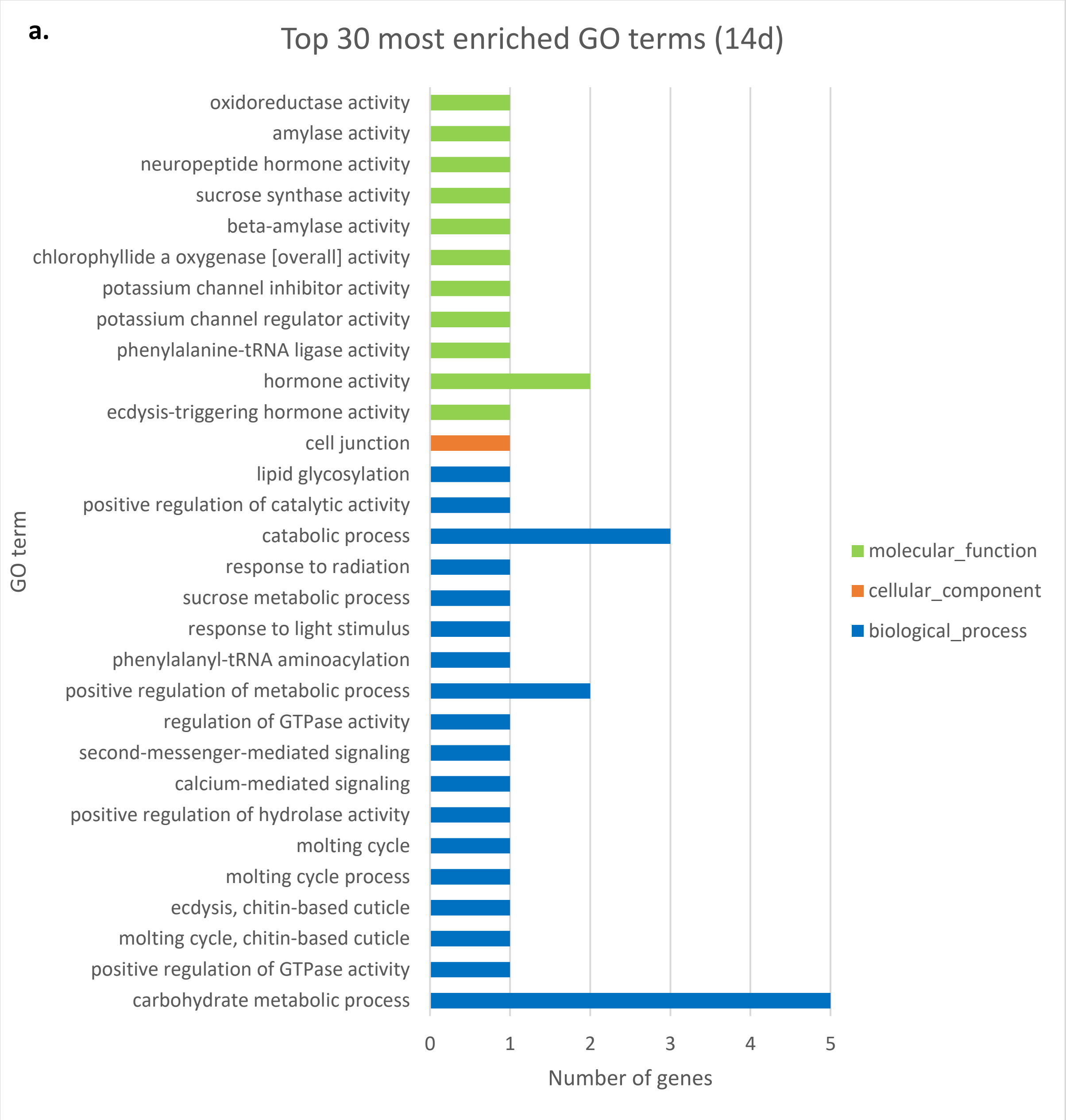

Additional file 2: Figure S1. Gene Ontology (GO) analysis of DEGs in the roots after 14d of phosphate deprivation treatment. The y- axis shows the enriched GO terms and the x-axis shows the number of DEGs. Green columns represent the molecular function. Orange columns represent the cellular component. Blue columns represent the biological process.

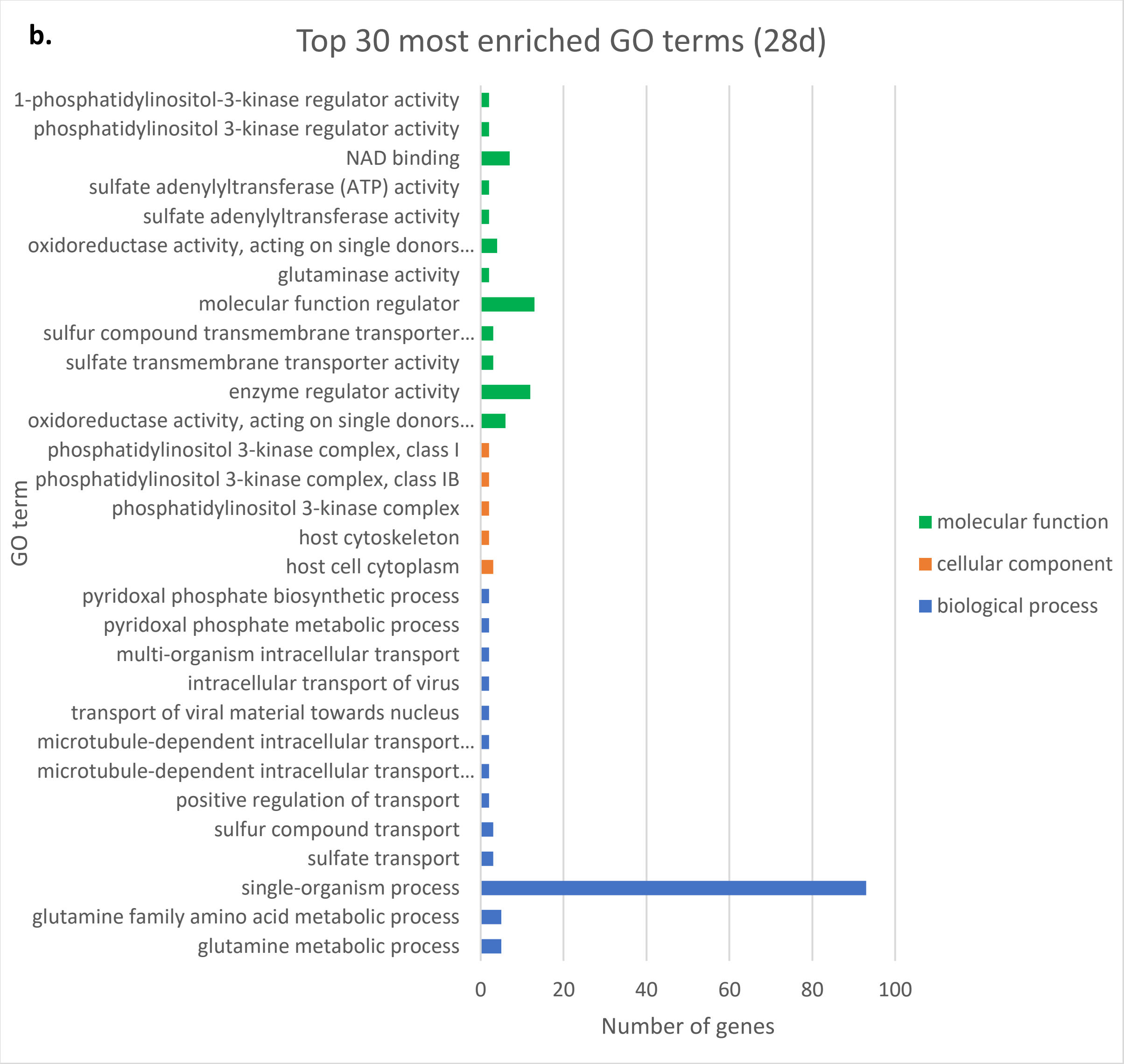

Additional file 2: Figure S2. Gene Ontology (GO) analysis of DEGs in the roots after 28d of phosphate deprivation treatment. The y- axis shows the enriched GO terms and the x-axis shows the number of DEGs. Green columns represent the molecular function. Orange columns represent the cellular component. Blue columns represent the biological process.
